# Wubi Shanyao Pills ameliorate glucocorticoid-induced osteoporosis by regulating bone marrow T cell homing as an immune intermediate to restore bone metabolism

**DOI:** 10.1186/s13020-026-01452-7

**Published:** 2026-07-02

**Authors:** Ying-Jie Dong, Jia-Yue Zhu, Jie Su, Ya-Wen Sun, Er-Xiao Chen, Xiao-Hu Jin, Gui-Yuan Lv, Su-Hong Chen

**Affiliations:** School of Pharmaceutical Sciences, Zhejiang Chinese Medical University, No. 548, Binwen Road, Binjiang District, Hangzhou, 310014 Zhejiang China

**Keywords:** Wubi Shanyao Pills (WBSY), Glucocorticoid-induced osteoporosis (GIOP), T cell homing, RANKL/RANK/OPG, Bone metabolism

## Abstract

**Background:**

The disruption of bone immune function and bone metabolism homeostasis is intricately linked to the onset and progression of glucocorticoid-induced osteoporosis (GIOP). From the perspective of indirect pharmacology, bone immune cells act as critical intermediate mediators that bridge systemic drug exposure and local bone tissue responses. Wubi Shanyao Pills (WBSY), a Chinese patent medicine, have been shown to modulate T lymphocyte levels in naturally aging mice and improve bone strength. However, whether WBSY exerts anti-GIOP effects through an indirect immunomodulatory pattern and its underlying intermediate mechanisms remain unclear.

**Purpose:**

This study aims to elucidate the effects and mechanisms of WBSY on GIOP in mice.

**Methods:**

Initially, the chemical constituents of WBSY were characterized using high-performance liquid chromatography (HPLC). Following this, a GIOP mouse model was developed through intraperitoneal administration of dexamethasone at a dosage of 10 mg/kg every three days. The mice were subsequently treated with WBSY at varying dosages—high (1.5 g/kg), medium (0.75 g/kg), and low (0.375 g/kg)—via gavage over a 20-week duration. Throughout the experimental period, general physiological parameters of the mice were systematically monitored. Bone mass alterations were subsequently assessed through bone strength measurements, micro-computed tomography analysis of bone tissue, and histopathological examination. Potential target pathways were identified via transcriptomic analysis. Furthermore, bone immunology-related parameters were evaluated using flow cytometry, including the proportion of T lymphocytes in the spleen and bone marrow, as well as the expression levels of the CXC chemokine ligand 10-chemokine receptor 3 (CXCL10-CXCR3) axis. Concurrently, key indicators of the bone metabolism pathway, such as the Receptor Activator of Nuclear Factor-κB (RANK), Receptor Activator of Nuclear Factor-κB ligand (RANKL), and osteoprotegerin (OPG), were further analyzed.

**Results:**

The results of the HPLC analysis demonstrated that the concentrations of echinacoside, acteoside, and schisandrol in WBSY were 7.82 mg/g, 2.18 mg/g, and 0.32 mg/g, respectively. Subsequently, the findings revealed that WBSY significantly enhanced general physiological parameters, including body weight, grip strength, and anal/back temperature, in GIOP mice. WBSY not only enhanced bone strength and mitigated pathological damage to the femur, but also significantly improved bone microarchitecture. This improvement was evidenced by an increase in bone volume/tissue volume, trabecular thickness, and trabecular number, alongside a reduction in trabecular separation and the structural model index. Furthermore, transcriptomic analysis indicated that WBSY may exert anti-GIOP effects by modulating key pathways, including osteoclast differentiation, interleukin-17A (IL-17A), and nuclear factor kappa B (NF-κB). At the immunological level, WBSY was shown to increase the proportion of T cells, B cells, and CD4⁺ T cells in the spleen, downregulate the expression of CXCL10 and CXCR3, and inhibit the activation of CD4⁺ Th17 cells in the femur. At the molecular level, WBSY modulated the expression of key molecules involved in the bone metabolism pathway, including RANK, RANKL, and OPG. It consistently downregulated the levels of femoral inflammation signaling molecules such as tumor necrosis factor receptor-associated factor 6 (TRAF6), phosphorylated IκB kinase β/IκB kinase β (p-IKKβ/IKKβ), and phosphorylated nuclear factor kappa B/nuclear factor kappa B (p-NF-κB/NF-κB). Additionally, WBSY reduced the concentrations of bone resorption markers, including tartrate-resistant acid phosphatase (TRAP) and β-C-telopeptide of type I collagen (β-CTX). Concurrently, it upregulated the expression of osteogenic molecules such as Runt-related transcription factor 2 (Runx2) and bone morphogenetic protein 2 (BMP2), leading to increased concentrations of bone formation markers, including bone-specific alkaline phosphatase (BALP), procollagen type I N-terminal propeptide (PINP), and osteocalcin (BGP).

**Conclusions:**

In summary, this study suggests that WBSY exerts anti-GIOP effects via an indirect pharmacological mechanism. WBSY may modulate the CXCL10-CXCR3 axis to inhibit aberrant T cell homing to the bone marrow, thereby reducing the excessive activation of bone marrow-resident Th17 cells and the secretion of the key bridging cytokine IL-17A. This indirect immunomodulation may subsequently restore the balance of the RANKL/RANK/OPG pathway, ultimately synergistically promoting bone formation and inhibiting bone resorption.

**Graphical Abstract:**

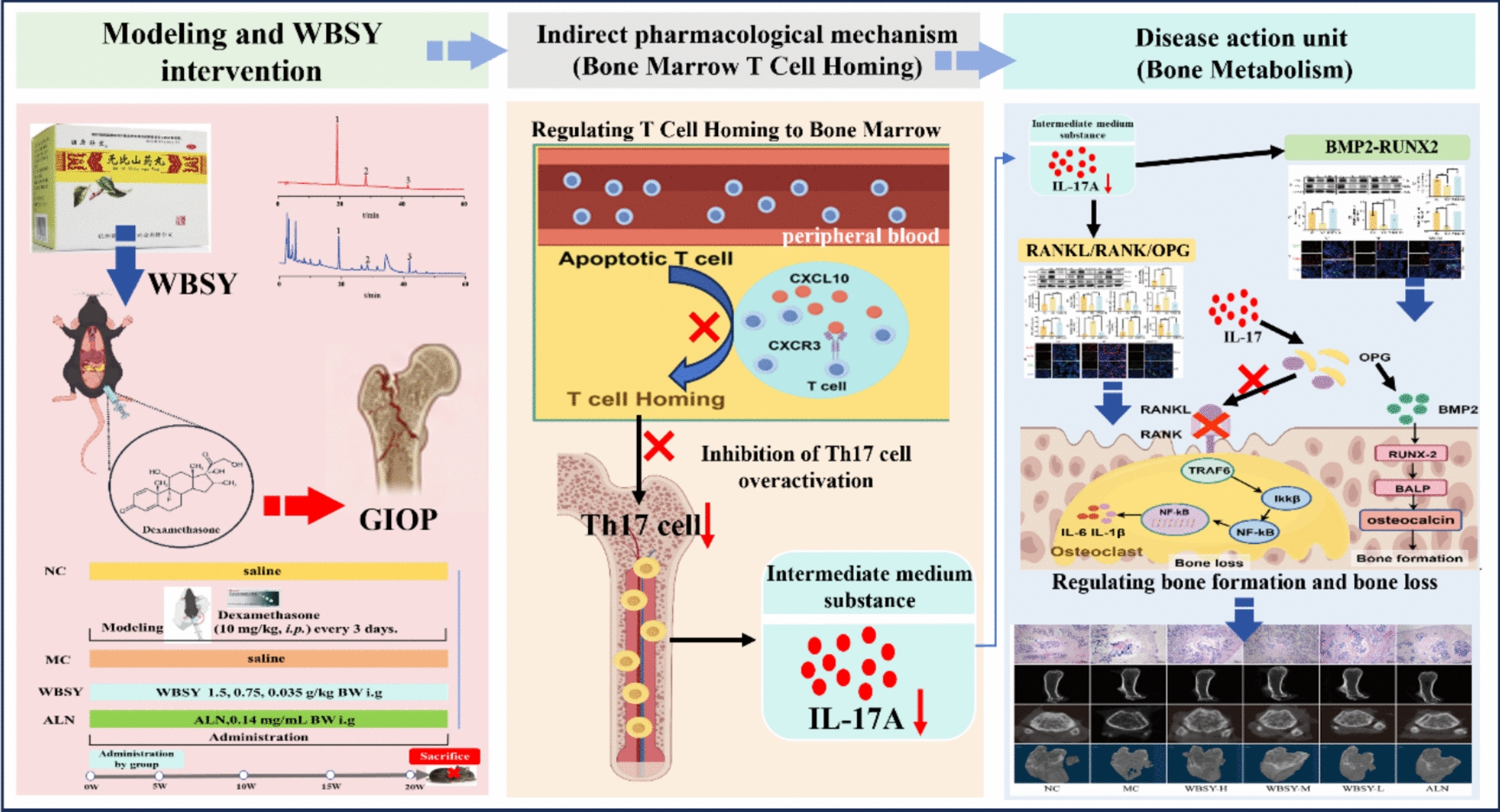

## Introduction

Osteoporosis is a systemic metabolic bone disorder characterized by the deterioration of bone microstructure and a reduction in bone mineral density. Contemporary research suggests that osteoporosis is multifactorial in origin, with contributing factors including glucocorticoid overuse, metabolic dysregulation, endocrine disorders, and estrogen deficiency [20]. Clinically, glucocorticoids are extensively utilized in the management of allergic, autoimmune, and post-transplant conditions [19]. Dexamethasone, a potent and long-acting synthetic glucocorticoid, is frequently employed for its significant anti-inflammatory and immunosuppressive properties. Nevertheless, GIOP is among the most serious and prevalent adverse effects associated with prolonged dexamethasone therapy. It constitutes the leading cause of secondary osteoporosis and iatrogenic fractures. Clinical studies report that the incidence rate of osteoporosis following glucocorticoid treatment exceeds 80%. Alarmingly, approximately one-third of affected patients do not receive standardized treatment, significantly compromising their quality of life [54]. Recent epidemiological data identify GIOP as the predominant cause of secondary osteoporosis, responsible for 20% of such cases [48]. Consequently, there is an urgent need for standardized prevention and treatment strategies, as well as a deeper understanding of the mechanistic pathways involved in GIOP pathogenesis [41].

The discipline of bone immunology underscores the pivotal involvement of T cells in the pathogenesis of GIOP [45]. Empirical evidence indicates that prolonged administration of dexamethasone can trigger apoptosis in peripheral T cells. However, a subset of these T cells migrates to the bone marrow microenvironment via a CXCL10-CXCR3 axis-dependent pathway, leading to their abnormal accumulation. This aberrant recruitment perturbs the local immune homeostasis and the balance of bone metabolism within the bone marrow, thereby impairing the normal regulatory function of the bone immune interface [24, 42]. Furthermore, under sustained dexamethasone stimulation, T cells retained in the bone marrow become overactivated, with a marked upregulation in the expression of Retinoic acid-related orphan receptor gamma (RORγt). As a key transcription factor for Th17 cell differentiation, RORγt activation directly facilitates the differentiation of naïve T cells into Th17 cell subsets, culminating in an anomalously elevated proportion of Th17 cells within the bone marrow [11, 13].

The excessive activation of Th17 cells exacerbates bone loss through a dual mechanism. Firstly, Th17 cells can stimulate osteoclast precursor cells to directly express RANKL, thereby accelerating osteoclastogenesis and bone resorption processes. This effect is corroborated by elevated levels of biomarkers such as TRAP and β-CTX [3, 7, 14]. Conversely, Th17 cells secrete cytokines associated with osteoclastogenesis, such as IL-17A, which are accompanied by inflammatory infiltration. The upregulation of NF-κB further enhances the expression of RANKL, thereby promoting osteoclastogenesis and inhibiting Runx2-mediated osteogenic processes, as evidenced by decreased levels of BALP, PINP, and BGP [28, 35, 49]. Ultimately, this leads to the degradation of the trabecular bone structure and a reduction in bone mechanical strength. Therefore, elucidating and targeting these specific T cell-driven pathways, particularly the Th17/IL-17A axis and its disruption of the RANKL/Runx2 balance, is essential for developing effective therapies and restoring bone immune homeostasis in patients with GIOP. The antagonistic effect of BMP2, through the enhancement of Runx2 activity, further underscores the potential of this therapeutic approach [8].

WBSY is a proprietary Chinese medicine approved by the National Medical Products Administration, frequently utilized in clinical settings to mitigate symptoms of lumbar and knee soreness and weakness. The formulation consists of twelve medicinal ingredients: the rhizome of *Dioscorea opposita* Thunb (Shanyao), processed products of *Rehmannia glutinosa* f. *lutea* Y.C.Chu & J.F.Li (Shudihuang), the bark of *Eucommia ulmoides* Oliv. (Duzhong), the fleshy stem of *Cistanche deserticola* var. *flabellata* R.Cao & Q.Ma (Roucongrong), the ripe fruit pulp of *Cornus officinalis* Siebold et Zucc. (Shanzhuyu), the sclerotia of *Poria cocos* (Schw.) Wolf (Fuling), mature seeds of *Cuscuta australis* R.Br. (Tusizi), the dry root of *Morinda officinalis* F.C.How (Bajitian), the dry tuber of *Alisma plantago-aquatica* L. (Zexie), the dry root of *Achyranthes bidentata* Blume (Niuxi), the ripe fruit pulp of S*chisandra hanceana* Baill. (Wuweizi), and *Halloysitum Rubrum* (Chishizhi). The plant nomenclature was verified against www.worldfloraonline.org, while the fungal and mineral names were confirmed with reference to the *Pharmacopoeia of the People's Republic of China (2025 Edition)*.

Recent research has increasingly clarified the pharmacological properties of key medicinal components in WBSY, particularly their potential roles in regulating bone metabolism and mitigating bone-related disorders. Research has demonstrated that *Dioscorea opposita* exhibits anti-osteoporotic effects by inhibiting bone resorption, increasing bone mass, enhancing bone hardness and strength, and repairing microfractures in trabecular bone and bone trabeculae [10]. Further investigations have indicated that *Cornus officinalis* exhibits therapeutic effects including combating oxidative stress and promoting bone tissue regeneration [1]. In the context of osteogenesis, S*chisandra hanceana* can significantly reduce the generation of osteoclasts induced by RANKL and ameliorate osteoporosis [18]. Previous studies have demonstrated that WBSY can promote osteoblast-mediated bone formation and inhibit osteoclast-mediated bone resorption, thereby ameliorating osteoporosis in postmenopausal mice [16]. Additionally, WBSY has been shown to improve T lymphocyte levels in aging model mice, further contributing to enhanced bone strength.

Indirect pharmacology is an emerging field focused on indirect drug action modes. Unlike conventional direct-acting agents such as alendronate, which directly suppresses osteoclast function, traditional Chinese medicines often exert systemic effects by modulating intermediate networks including immune, endocrine, and metabolic networks. In bone diseases, bone immune cells serve as critical mediators linking systemic intervention to local remodeling, and targeting these cells has emerged as a promising osteoporosis treatment strategy. Previous work has shown that WBSY regulates T lymphocyte homeostasis and improves bone strength in naturally aged mice. We therefore hypothesize that WBSY ameliorates GIOP through an indirect immunomodulatory mechanism, whereby it corrects aberrant bone marrow T cell homing and differentiation as the central intermediate step to restore bone metabolic balance. To test this hypothesis, we established a GIOP mouse model via 20-week intraperitoneal dexamethasone administration with concurrent WBSY treatment. We first evaluated WBSY's systemic and skeletal protective effects by measuring general physiological parameters, bone strength and trabecular microarchitecture. We then quantified the CXCL10-CXCR3 axis expression and bone marrow T cell homing, the key intermediate events. Finally, we examined downstream Th17 cell polarization, IL-17A secretion and the RANKL/RANK/OPG axis to delineate the underlying indirect pharmacological mechanism.

## Materials and methods

### Materials

WBSY (0.075 g/pill, 9 g/bag, 2313904, 2013910, 2013913) was provided by Hangzhou Hu Qing Yu Tang Pharmaceutical Co., Ltd. (Zhejiang, China). Dexamethasone (2206112211) was provided by Chenxin Pharmaceutical Co., Ltd. (Shandong, China). The PINP (WO07VB0R5982), BALP (WO08B2F87552) and BGP (WO06RON4542) reagent kits were provided by Wuhan Eilurete Biotechnology Co., Ltd. (Hubei, China). The -CTX assay kit (202404) was provided by Quanzhou Ruixin Biotechnology Co., Ltd. (Fujian, China). The TRAP (202405), CXCL10 (202406) and CXCR3 (202406) reagent kits were provided by Jiangsu Enzyme Immunity Industrial Co., Ltd. (Jiangsu, China). The FITC Rat Anti-Mouse CD45 (3145645), BV421 Hamster Anti-Mouse CD3e (3159687), APC Rat Anti-Mouse CD4 (3020927), PerCP-Cy5.5 Rat Anti-Mouse CD8a (3254772), PE-Cy7 Rat Anti-Mouse CD19 (3222461), PE Mouse Anti-Mouse NK-1.1 (3116554), FITC Rat Anti-Mouse IFN-γ (2010412), PE-Cy7 Rat Anti-Mouse IL-4 (3065878), PE Rat Anti-Mouse IL-17A (3160516) were purchased from BD Pharmingen Co., Ltd. (New Jersey, USA).

The antibodies for RANK (H651908019), RANKL (H680181017), OPG (H661760001) and TRAF6 (H651210041) were purchased from Hangzhou Huaan Biotechnology Co., Ltd. (Zhejiang, China). The antibodies for Runx2 (00144277), RORγt (00139487), CD4 (00130135), IL-17A (00129061), NF-κB p65 (00170304), IKKβ (00136757), BMP2 (10027024), Interleukin-1 Beta (IL-1β) (00132317) and Interleukin-6 (IL-6) (00141276) were purchased from Wuhan Sanying Biotechnology Co., Ltd. (Hubei, China). The Alkaline phosphatase stain solution (JR255216) was purchased from Yuanye Biotechnology Co., Ltd. (Shanghai, China). The antibody for p-IKKβ (lz48175) was purchased from Affinity Biosciences (Melbourne, USA). The Tartrate-resistant acid phosphatase dye solution (20240607) was purchased from Nanjing Jiacheng Technology Co., Ltd. (Jiangsu, China).

### Component analysis of WBSY by HPLC

The content determination of main components in WBSY was performed in accordance with the method specified in the *Pharmacopoeia of the People's Republic of China (2025 Edition)*. Briefly, approximately 2 g of powdered WBSY was accurately weighed, mixed with 25 mL of 50% methanol, and the total weight was recorded. The mixture was subjected to ultrasonic treatment for 30 min, allowed to cool to room temperature, and reweighed; the lost weight was replenished with 50% methanol. After shaking uniformly and filtration, the subsequent filtrate was collected as the test sample solution. For the reference solution, appropriate amounts of echinacoside, acteoside, and schisandrol reference substances were accurately weighed and dissolved in 50% methanol to prepare a mixed reference solution containing 400 μg/mL of echinacoside, 80 μg/mL of acteoside, and 10 μg/mL of schisandrol. HPLC analysis was carried out on an Agilent 1200 HPLC system (No. DE62962036) equipped with an Ultimate LP-C18 column (4.6 × 250 mm, 5 μm). The mobile phase consisted of acetonitrile (phase A) and 0.3% phosphoric acid solution (phase B) with a gradient elution program: 10–20% A (0–30 min), 20–55% A (30–35 min), and 55% A (35–50 min). The column temperature was maintained at 25 °C, the flow rate was 1.0 mL/min, the detection wavelength was set at 245 nm, and the injection volume was 10 μL. In order to further investigate the stability of the method, three different batches of WBSY (2013904, 2013910, 2013913) were taken for the above treatment. The test solution of each batch was injected continuously three times, and the common chromatographic peak areas were measured and compared for similarity.

### Animals and experiment design

48 male C57BL/6 mice, 8 weeks old, weighing 22 ± 2 g were obtained from the Hangzhou Qizhen Experimental Animal Technology Co., Ltd. (The license number 20231227Abzz0600080943). The animals were kept in a constant room (23 ± 2 °C, 55% ± 5% humidity), with 12 h light/12 h darkness, good ventilation and no large noise interference. Adaptive feeding for 7 days, free access to food and water. This study received approval from the Ethics Committee of Zhejiang Chinese Medical University (IACUC-202408-15). This study was conducted and reported in full compliance with the ARRIVE (Animal Research: Reporting of In Vivo Experiments) guidelines.

After an adaptation period of seven days, all mice were randomly divided into 6 groups (*n* = 8). They were normal control group (NC), model control group (MC), WBSY of high dose group (WBSY-H, 1.5 g/kg), WBSY of medium dose group (WBSY-M, 0.75 g/kg), WBSY of low dose group (WBSY-L, 0.375 g/kg), and Alendronate Sodium (ALN) positive group (ALN, 0.14 mg/kg). The dosages for WBSY were determined based on prior experimental findings [16]. Then, according to literature reports [26, 43], the MC group and the administration group received intraperitoneal injections of DEX at a dosage of 10 mg/kg, while the NC group of mice was administered physiological saline via intraperitoneal injection, with a frequency of once every three days. Concurrently, the corresponding drugs were also administered by gavage once daily for a duration of 20 weeks, with a gavage volume set at 0.1 mL/10 g, as illustrated in Fig. 2A. The NC group was provided with standard feed and allowed free access to water. On the last day of administration, blood was collected, and the mice were euthanized by cervical dislocation. The femurs and tibias were excised and stored at − 80 °C.

### Determination of physical indicators

Mice were weighed every two weeks, and the values were recorded. After 16 weeks of modeling and administration, the grip strength, rectal temperature, dorsal temperature, and locomotor activity counts of mice were measured in a quiet room environment at a temperature of (25 ± 2) ℃ and humidity of (55 ± 5)%.

### Determination of bone mass related indicators

After 20 weeks of modeling and administration, the left tibia of mice were harvested to assess bone strength using a small animal bone strength testing device. The right hind limb femur from each group of mice was fixed in 10% neutral formalin for two weeks. Subsequently, micro-computed tomography scanning was performed on the right femur to obtain reconstructed images for quantitative analysis [59]. This analysis provided parameters related to the microstructure of trabecular bone at the distal end of the femur: bone volume/tissue volume, trabecular thickness, trabecular number, trabecular separation and the structural model index.

### Enzyme-linked immunosorbent assay

After 20 weeks of administration, the levels of TRAP, β-CTX, BALP, PINP, BGP, CXCL10 and CXCR3 in serum were detected by enzyme-linked immunosorbent assay.

### Histopathological examination

H&E staining [53] was performed according to our previous study. As described in our earlier study [29], TRAP staining was utilized to assess the quantity of osteoclasts. Furthermore, as reported in recent literature [56], Alkaline Phosphatase (ALP) was employed to measure the levels of alkaline phosphatase within the femur. All histopathological changes were observed under a biological microscope (OLYMPUS BX43, Japan).

### Transcriptomic analysis

Total RNA was extracted from femoral tissue utilizing the Trizol reagent. In accordance with the standard protocol for RNA library preparation, 1 μg of total RNA was employed to construct RNA-seq transcriptome libraries. Messenger RNA (mRNA) was subsequently isolated through poly(A) selection using oligonucleotide (dT) beads and fragmented with lysis buffer. The process of cDNA synthesis, end repair, A-base addition, and ligation of index adapters was then carried out. A library size selection for cDNA target fragments ranging from 200 to 300 base pairs was performed using 2% low-range ultra-agarose, followed by PCR amplification involving 15 cycles with Phusion DNA polymerase. The libraries were quantified using TBS380 and sequenced in paired-end 150 mode on a next-generation sequencing platform MGI-T7 provided by Shanghai BIOSZERON Biotechnology Co., Ltd., following the standard protocol. Differentially expressed genes (DEGs) were identified using the DESeq software with a screening threshold of |log2FoldChange|> 1 and a false discovery rate (FDR) of less than 0.05. Enrichment analysis of the Gene Ontology (GO) and Kyoto Encyclopedia of Genes and Genomes (KEGG) pathways was conducted using the edgeR package, with pathways exhibiting a p-value of less than 0.05 considered significantly enriched.

### Flow cytometry

Flow cytometry analysis of immune cell subsets in spleen and bone marrow reference literature [57]. Following euthanasia and disinfection with 75% ethanol, spleens were harvested and mechanically dissociated in phosphate-buffered saline (PBS). Erythrocytes were lysed using ACK Lysing Buffer (Thermo Fisher Scientific, USA), and cell concentration was adjusted to 1 × 10^6^ cells/mL with an XT-2000i hematology analyzer (Sysmex, Japan). For surface staining, cells were incubated with FVS780 live/dead stain and fluorochrome-conjugated antibodies against CD45, CD3e, CD4, CD8a and CD19 for 30 min at 4 °C in the dark. Concurrently, bone marrow cells were flushed from femurs and tibias with ice-cold PBS, filtered through a 70-μm cell strainer, and adjusted to 1 × 10^6^ cells/mL. Cells were then incubated with FVS780 live/dead stain and fluorochrome-conjugated antibodies against CD3e, CD4 and CD8a for 30 min at 4 °C in the dark for surface staining. After surface staining, cells were fixed and permeabilized using a Fixation/Permeabilization Kit (BD Biosciences, USA) according to the manufacturer’s instructions, followed by intracellular staining with antibodies against IFN-γ, IL-4 and IL-17A for 30 min at 4 °C in the dark. All samples were acquired on a BD FACSCanto II flow cytometer (BD Biosciences, USA) and analyzed using FlowJo software. The gating strategy for immune cell subset identification was as follows: lymphocytes were first gated based on FSC-A versus SSC-A, single cells were selected using FSC-A versus FSC-H to exclude doublets, live cells were identified by exclusion of FVS780-positive dead cells, and finally immune subsets were defined within the live cell population as CD3⁺ T cells (CD45⁺CD3⁺), CD19⁺ B cells (CD45⁺CD19⁺), CD4⁺ T cells (CD45⁺CD3⁺CD4⁺), CD8⁺ T cells (CD45⁺CD3⁺CD8⁺), Th1 cells (CD45⁺CD3⁺CD4⁺IFN-γ⁺), Th2 cells (CD45⁺CD3⁺CD4⁺IL-4⁺) and Th17 cells (CD45⁺CD3⁺CD4⁺IL-17A⁺).

### Immunohistochemistry and immunofluorescence staining

As reported in the literature [38], the expression of CXCL10, CXCR3, IL-6 and IL-1β proteins in Femur tissues were determined by immunohistochemistry. Sections of Femur was taken at 4 μm, and corresponding antibodies were incubated, and then HRP conjugated goats were added to anti-rabbit IgG. The signal was displayed by DAB staining, and the nucleus was stained with hematoxylin. Finally, Image-Pro Plus software was used for semi-quantitative analysis of protein levels.

As we previously described [27], the femur was first stained with primary antibodies such as CD4, RORγt, IL-17A, RANK, RANKL, OPG, TRAF6, NF-κB p65, IKKβ, Runx2, and BMP2. AffiniPure Goat Anti-rabbit IgG (H + L) was coupled with FITC, then stained with DAPI, and sealed with anti-fluorescence quenching reagent. Fluorescence electron microscope was used to photograph the expression. The green fluorescence signifies the positive expression of the RORγt, OPG, NF-κB p65, and BMP2 antibodies, the red fluorescence signifies positive expression of the CD4, RANKL, TRAF6, and Runx2 antibodies, the purple fluorescence represents positive expression of the IL-17A, RANK, and IKKβ antibodies, while the blue fluorescence was the expression of the nucleus.

### Western blot analysis

According to the previous method [52], western blot was used to detect the expression of femoral RANK/RANKL/OPG pathway, femoral TRAF6- IKKβ-NF-κB pathway and RUNX2/BMP2 pathway. In short, the femur was placed in pre-cooled RIPA buffer containing PMSF and lysed at 4 °C for 1 h. The femoral tissues were homogenized, centrifuged at 12,000 r/min for 10 min, and the supernatant was collected. The protein concentration of femoral tissue was determined by BCA method. The protein samples of femoral tissue were separated by 10% SDS-PAGE and transferred to PVDF membrane. The protein samples were incubated with the corresponding antibodies. After that, the membrane was incubated in a suitable secondary antibody conjugated with HRP for 2 h, and then washed 3 times in TBST solution. Finally, the protein bands were displayed by the ECL kit and analyzed by the Image-J software.

### Quantitative real-time polymerase chain reaction (qRT-PCR)

As we have studied before [30], Total RNA was isolated from the femur using TRIzol & Plus RNA Purification Kit (Invitrogen) and RNase-Free DNase Reagent (Qiagen). Reverse transcription was then performed using the SuperScriptTM III First-Strand Synthesis SuperMix kit (Invitrogen) for qRT-PCR. The mRNA expression of *CXCL10*, *CXCR3*, *TRAF6*, *NF-κB*, *IKKβ*, *CD4*, *RORγt*, *IL-17A*, *IL-6*, *IL-1β*, *RANK, RANKL* and *OPG* in femoral were detected by qRT-PCR using CFX384 Touch qRT-PCR detection system (Bio-Rad, USA). Normalize the relative expression levels using the internal reference gene (*GAPDH*). The primer sequence is shown in Table 1.

**Table 1:** Quantitative real-time PCR primers

Genes	Sequences (5ʹ−3ʹ)	GeneBank
*Actin*	Forward: CACTGTCGAGTCGCGTCC	NM_007393.5
	Reverse: CGCAGCGATATCGTCATCCA	
*CXCL10*	Forward: CATCCTGCTGGGTCTGAGTG	NM_001565.4
	Reverse: AGCTTCCCTATGGCCCTCAT	
*CXCR3*	Forward: TACCTTGAGGTTAGTGAACGTCA	NM_001504.2
	Reverse: CGCTCTCGTTTTCCCCATAATC	
*CD4*	Forward: CCAGACAGTGTTCCTGGCTT	NM_013488.3
	Reverse: TGCCTGGCGCTGTTGG	
*RoR*-*γt*	Forward: TACCCTACTGAGGAGGACAGG	NM_001293734.1
	Reverse: AACCCCGTAGTGGATCCCAG	
*IL-17A*	Forward: TCCACCGCAATGAAGACCCT	NM_010552.3
	Reverse: CATGTGGTGGTCCAGCTTTCC	
*TRAF6*	Forward: AGTGCCCAGTTGACAATGAAA	NM_009424.3
	Reverse: CACTTTACCGTCAGGGAAAGAAT	
*NF-κB*	Forward: GCACCAAGACGGAACCCATC	NM_008689.3
	Reverse: GCGTAGTCGAAAAGGGCGTT	
*IKKβ*	Forward: AAGAACAGAGACCGCTGGTG	NM_001159774.2
	Reverse: ACAACGATGTCCACTTCGCT	
*IL-6*	Forward: CCCCAATTTCCAATGCTCTCC	NM_031168.2
	Reverse: CGCACTAGGTTTGCCGAGTA	
*IL-1β*	Forward: GCTTCAGGCAGGCAGTATCA	NM_010552.3
	Reverse: AATGGGAACGTCACACACCA	
*RANK*	Forward: CGGCGTTTACTACAGGAAGGG	NM_009399.4
	Reverse: CTTCTTGCTGACTGGAGGTTGC	
*RANKL*	Forward: CCATCGGGTTCCCATAAAGTCA	NM_011613.3
	Reverse: CAGTTTTTCGTGCTCCCTCCTT	
*OPG*	Forward: CCCTTGCCCTGACCACTCTTAT	NM_008764.3
	Reverse: AACTGTGTTTCGCTCTGGGGTT	

### Statistical analysis

All data were analyzed by one-way analysis of variance, expressed as mean ± standard deviation (SD). The results were considered statistical significance at *p* < 0.05. Graphs were plotted using GraphPad Prism 8 software.

## Results

### Component of WBSY

**Fig. 1:**
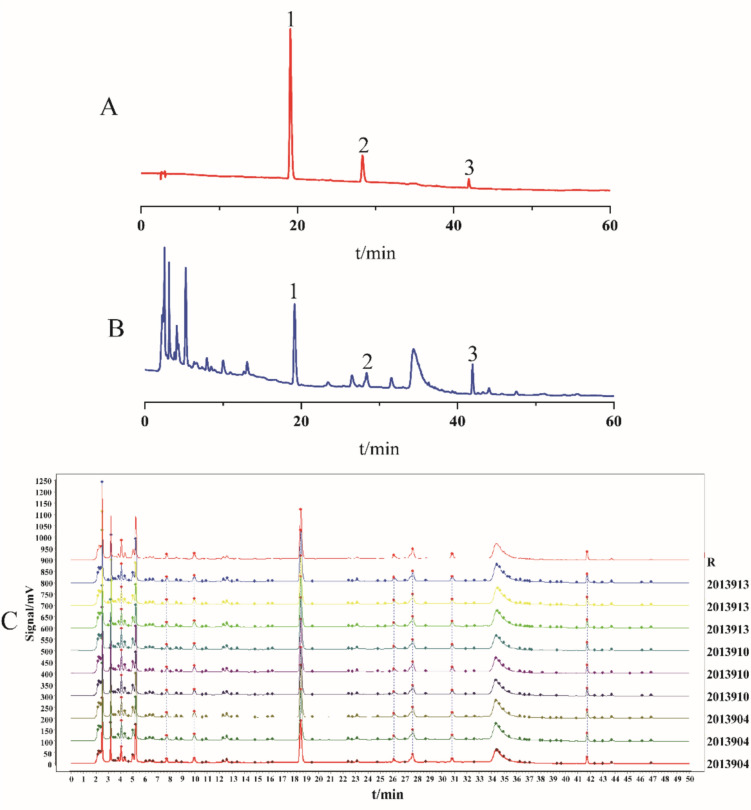
HPLC chromatogram of WBSY. Echinacoside (1), acteoside (2), and schisandrol (3) detected in reference solution (**A**), and in the WBSY (**B**). Fingerprint analysis (**C**)

As shown in Fig. 1A and B, the HPLC analysis results indicated that the contents of echinacoside, acteoside, and schisandrol in WBSY were 7.82 mg/g, 2.18 mg/g, and 0.32 mg/g, respectively. According to the requirements specified in the Pharmacopoeia of the *Pharmacopoeia of the People's Republic of China (2025 Edition)*, the total content of echinacoside and acteoside per 1 g of WBSY shall not be less than 2.5 mg, and the content of schisandrol shall not be less than 0.10 mg. All the above-mentioned component contents in the sample were significantly higher than the limits stipulated by the Pharmacopoeia, which is consistent with our previous research findings [16]. To further assess the stability of the WBSY, three test samples were obtained from different batches and subjected to triplicate injections. The chromatographic analysis revealed a similarity index exceeding 0.999, while the RSD values of the relative peak areas for each common peak were below 3%. These findings indicate a high degree of drug stability, as shown in Fig. 1C, Table 2.

**Table 2:** Stability similarity results

Batch number	2013904	2013904	2013904	2013910	2013910	2013910	2013913	2013913	2013913	RSD(%)
Reference fingerprint spectrum R	1	1	1	0.999	0.999	0.999	1	1	1	0.1

### Effects of WBSY on physical indicators of model mice

**Fig. 2:**
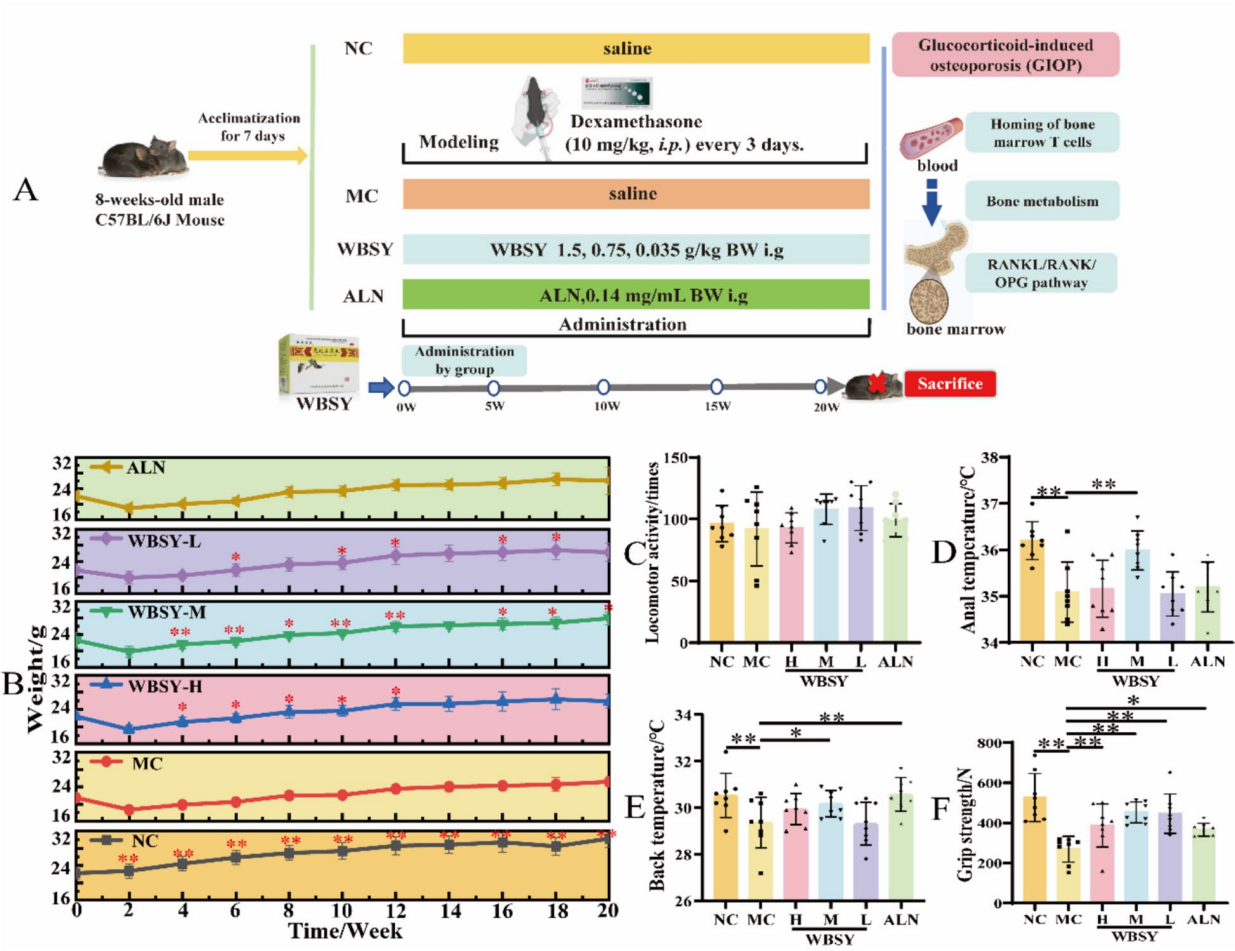
Effects of WBSY on physical indicators of model mice. Schematic diagram of the experimental design (**A**), Physiological parameters body weight (**B**), locomotor activity (**C**), anal temperature (**D**), back temperature (**E**), grip strength (**F**) in mice, *n* = 8. Data are expressed as the mean ± SD. *p < 0.05 and **p < 0.01 compare with the model control group

Long term use of Dexamethasone can reduce the weight of model mice. Our study demonstrated that, compared to the NC, the MC exhibited a significant reduction in body weight from the 2nd week (*p* < 0.01), which persisted until the 20th week (*p* < 0.01). In contrast, WBSY-M significantly increased body weight from the 4th week onward (*p* < 0.01), continuing until the 20th week (*p* < 0.05), as illustrated in Fig. 2B. Grip strength, anal temperature, and back temperature serve as key physiological indicators in mice. Unexpectedly, the MC showed markedly decreased grip strength, anal temperature, and back temperature after 16 weeks of dexamethasone administration (*p* < 0.01). All treatment groups significantly restored grip strength (*p* < 0.05, *p* < 0.01), while the MBSY-M additionally elevated anal and back temperatures (*p* < 0.05, *p* < 0.01). Data are presented in Fig. 2C–F.

### Effects of WBSY on bone microstructure of model mice

**Fig. 3:**
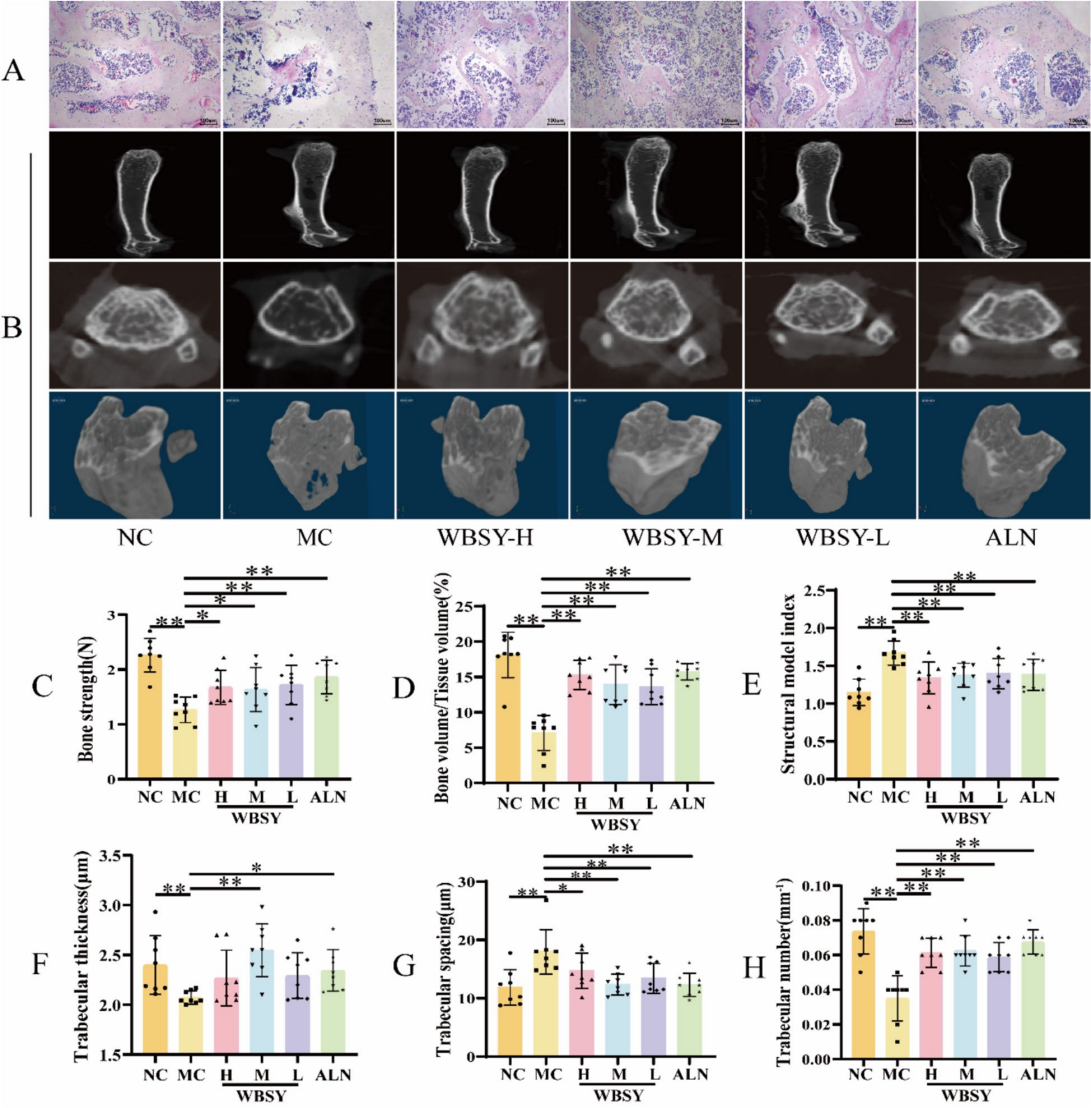
Effects of WBSY on Bone microstructure of model mice. **H**&**E** staining (SP × 200) of Femur (**A**), representative Micro Computed Tomography image of the distal femoral metaphysis in mice (**B**), femoral Bone Strength in Mice (**C**), quantification of trabecular bone microarchitecture parameters from Micro Computed Tomography analysis in mouse femur: Bone volume/tissue volume ratio (**D**), structural model index (**E**), trabecular thickness (**F**), trabecular spacing (**G**) and trabecular number (**H**), *n* = 8. Data are expressed as the mean ± SD. ^*^p < 0.05 and ^**^p < 0.01 compare with the model control group

Bone strength serves as a critical indicator of bone integrity and functionality. In comparison to the NC group, the bone strength in the MC group was significantly diminished (*p* < 0.01). Histological analysis using H&E staining revealed that the femoral trabeculae in the MC group were slender and sparsely arranged, exhibiting reduced connectivity, increased interstitial spaces, significant fractures, and markedly enlarged bone marrow cavities. Conversely, as illustrated in Fig. 3A and C, treatment with WBSY significantly enhanced bone strength (*p* < 0.05, *p* < 0.01), improved trabecular morphology, increased trabecular density and continuity, and inhibited the expansion of the medullary cavity.

Micro-computed tomography imaging (Fig. 3B) corroborated these observations, revealing sparse, fractured, and discontinuous trabeculae in the MC group. In contrast, treatment with WBSY restored trabecular volume and connectivity, resulting in a denser and more complete trabecular network. Quantitative data analysis further substantiated the bone protective effects of WBSY. The MC group exhibited a significant reduction in bone volume/tissue volume ratio, trabecular thickness, and trabecular number (*p* < 0.01), alongside an increase in the structural model index and trabecular spacing (*p* < 0.01), as described in Fig. 3D–H. However, administration of WBSY reversed these alterations. These findings indicate that WBSY effectively mitigates bone loss in osteoporosis models by enhancing trabecular morphology, and increasing bone mass and strength, with WBSY-M demonstrating superior efficacy.

### Analysis of femur transcriptomics

**Fig. 4:**
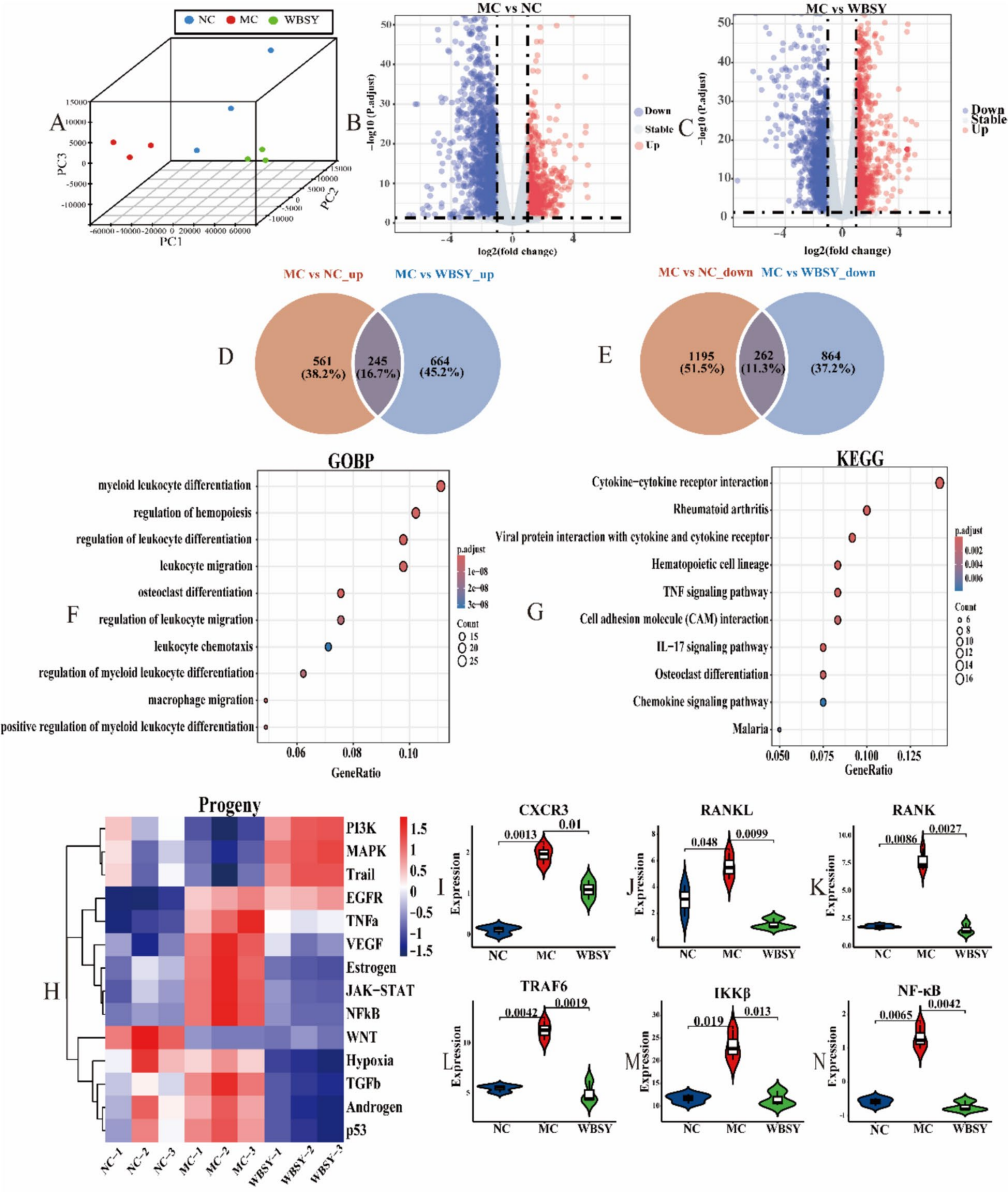
Transcriptome sequencing analysis of differentially expressed genes and enriched pathways in the femur of model mice. 3D Principal Component Analysis (**A**), volcano Plot of the differential gene (**B**, **C**), venn diagram of the differential gene (**D**, **E**), GOBP (**F**), KEGG (**G**), progeny_heatmap (**H**), comparison of differences in the construction of *CXCR3, RANKL, RANK, TRAF6, IKK β,* and *NF-κ B* genes (**I**–**N**), *n* = 3. Data are expressed as the mean ± SD. ^*^p < 0.05 and ^**^p < 0.01 compare with the model control group

Principal component analysis was employed to assess the extent of sample dispersion, with shorter distances between sample points indicating greater compositional similarity. As illustrated in Fig. 4A, the WBSY samples exhibited closer clustering with the normal control group. Differential gene expression analysis was performed using DESeq software, applying screening criteria of |log2FC|> 1 and FDR < 0.05 for significance. The findings, depicted in Fig. 4B–E, revealed that, relative to the normal control group, 806 genes were upregulated and 1457 genes were downregulated in the model control group. In comparison to the model control group, the WBSY group demonstrated upregulation of 909 genes and downregulation of 1126 genes.

An enrichment analysis was conducted utilizing the number of enriched genes and their corresponding P-values. The bubble chart from the GO enrichment analysis illustrated that DEGs were predominantly enriched in biological processes such as osteoclast differentiation, leukocyte migration, and muscle contraction (refer to Fig. 4F). Similarly, the bubble chart from the KEGG enrichment analysis revealed that DEGs were chiefly enriched in pathways including the IL-17 signaling pathway, osteoclast differentiation pathway, and chemokine signaling pathway (refer to Fig. 4G). Additionally, the progeny method was employed to assess the activity of relevant signaling pathways, with the results visualized through heatmaps and subjected to intergroup statistical analyses. The findings indicated a significant downregulation in the expression of key genes such as *CXCR3, RANKL, RANK, TRAF6, NF-κB* and *IKKβ* following WBSY intervention, as shown in Fig. 4H–N. In summary, WBSY may counteract GIOP by modulating the activity of critical pathways, including those involved in osteoclast differentiation, IL-17 signaling, and NF-κB.

### Effect of WBSY on the homing of bone marrow T cells in model mice

**Fig. 5:**
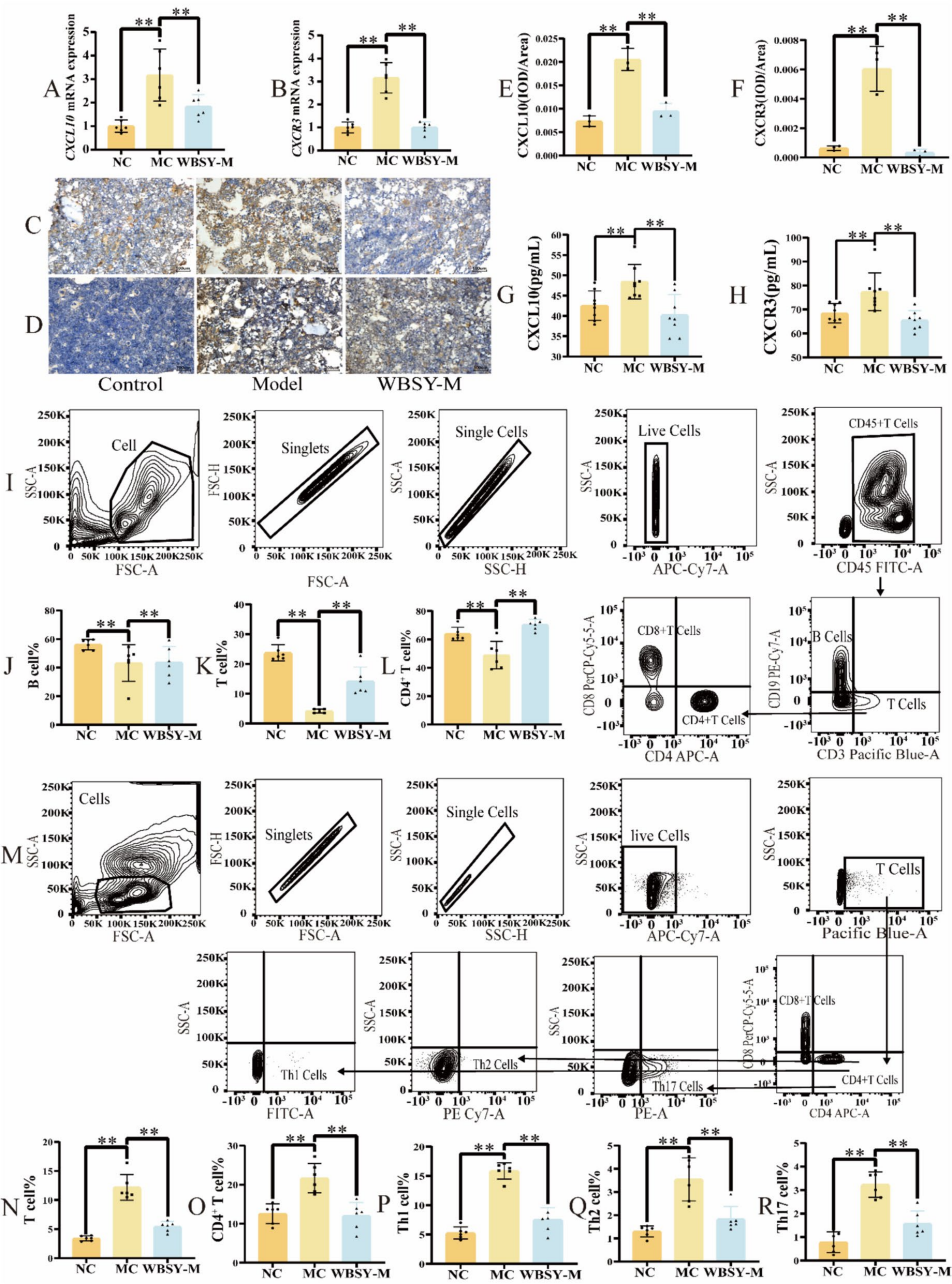
Effect of WBSY on the homing of bone marrow T cells in model mice. The mRNA expression of *CXCL10* and *CXCR3* in the femur (**A**, **B**), *n* = 6. The expression of CXCL10 and CXCR3 in femur were observed by immunohistochemistry at 400 × magnification, and the IOD/Area was counted by Imagine Pro (**C**–**F**), *n* = 3. Serum CXCL10 (**G**) and CXCR3 (**H**) levels, *n* = 8. Representative flow cytometry gating strategy diagrams depicting splenic T lymphocytes, B lymphocytes, and CD4⁺ T lymphocytes (**I**). Statistical analysis of T lymphocytes% (**J**), T lymphocytes% (**K**), and CD4⁺ T lymphocytes% (**L**). Representative flow cytometry gating strategy diagrams illustrating bone marrow T lymphocytes, CD4⁺ T lymphocytes, Th1 cells, Th2 cells, and Th17 cells (**M**). Statistical analysis of T lymphocytes% (**N**), CD4⁺ T lymphocytes% (**O**), Th1 cells% (**P**), Th2 cells% (**Q**), and Th17 cells% (**R**), n = 6. Data are expressed as the mean ± SD. ^*^p < 0.05 and ^**^p < 0.01 compare with the model control group

To validate the transcriptomic findings, it was observed that the expression of the *CXCR3* gene in GIOP model mice exhibited significant abnormalities. qRT-PCR was employed to assess the expression levels of pertinent genes, revealing a marked upregulation of *CXCR3* and *CXCL10* transcription levels in the MC group (*p* < 0.01). To further elucidate the changes in protein expression associated with the CXCR3 signaling pathway, immunohistochemical staining of femoral tissue and enzyme-linked immunosorbent assay of serum were conducted to evaluate the expression of CXCL10 and CXCR3, respectively. The findings indicated a significant elevation in serum levels of CXCL10 and CXCR3 in the MC group (*p* < 0.01), alongside a notable increase in the positive expression rates of these proteins in femoral tissue (*p* < 0.01). Notably, after a 20-week intervention with WBSY, the aberrant overexpression of CXCR3 and CXCL10 genes and proteins in the femur was significantly attenuated (*p* < 0.01), and the serum levels of CXCR3 and CXCL10 were effectively reduced (*p* < 0.01), depicted in Fig. 5A–H.

Building on these findings, the present study further employed flow cytometry to examine alterations in the proportions of T cell subsets within the spleen and bone marrow. The data (Fig. 5I–R) revealed that, in comparison to the NC group, the MC group exhibited a significant reduction in the proportions of T lymphocytes, B lymphocytes, and CD4^+^ T lymphocytes in the spleen (*p* < 0.01). Conversely, there was a significant increase in the proportions of T lymphocytes, CD4^+^ T lymphocytes, Th1 cells, Th2 cells, and Th17 cells in the bone marrow (*p* < 0.01). Treatment with WBSY-M was found to significantly ameliorate these aberrant changes in the previously described e cell subpopulations (*p* < 0.01).

### Effects of WBSY on RANKL/RANK/OPG pathway in bone of model mice

**Fig. 6:**
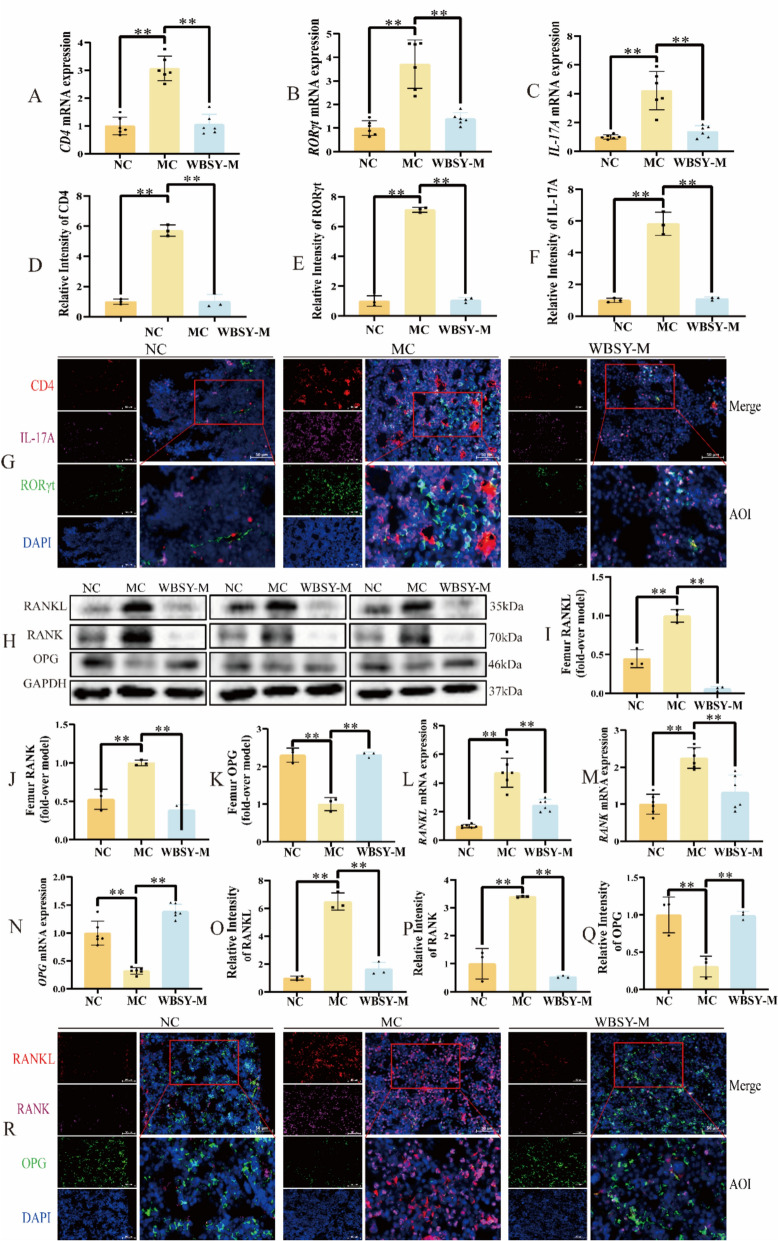
Effects of on RANKL/RANK/OPG pathway in bone of model mice. The mRNA expression of *CD4*, *RORγt* and *IL-17A* in the femur (**A**–**C**), n = 6. Immunofluorescence staining of CD4, RORγt, and IL-17A in femur and femur representative microscopic images were counted by Image-J (400 ×) (**D**–**G**), n = 3. The femur RANKL/RANK/OPG western blot representation (**H**) and protein expression statistical analysis (**I**–**K**), n = 3. The mRNA expression of *RANKL*, *RANK* and *OPG* in the femur (**L**–**N**), n = 6. Immunofluorescence staining of RANKL, RANK and OPG in femur and femur representative microscopic images were counted by Image-J (400 ×) (**O**–**R**), n = 3. Data are expressed as the mean ± SD. ^*^p < 0.05 and ^**^p < 0.01 compare with the model control group

To further substantiate the transcriptome findings and elucidate the underlying mechanism of WBSY's bone protective effects, we examined its influence on Th17 cell differentiation and the RANKL/RANK/OPG signaling axis. qRT-PCR analysis (Fig. 6A–C) demonstrated that, in comparison to the NC group, the MC group exhibited a significant upregulation in the mRNA expression levels of femoral *CD4*, *RORγt*, and *IL-17A* (*p* < 0.01). However, treatment with WBSY-M effectively inhibited these increases. Immunofluorescence staining (Fig. 6D–G) further corroborated that MC mice displayed a higher prevalence of CD4⁺ RORγt⁺ IL-17A⁺ cells. Concurrently, western blot (Fig. 6H–K) and qRT-PCR analyses (Fig. 6L–N) revealed that the MC group showed elevated protein and mRNA levels of RANK and RANKL (*p* < 0.01), alongside a reduction in OPG expression (*p* < 0.01), whereas WBSY-M treatment successfully reversed these alterations (*p* < 0.01). Immunofluorescence staining (Fig. 6O–R) confirmed the observed changes in the expression of RANKL, RANK, and OPG within bone tissue (*p* < 0.01) , aligning with our molecular data.

### Effects of WBSY on bone resorption of model mice

**Fig. 7:**
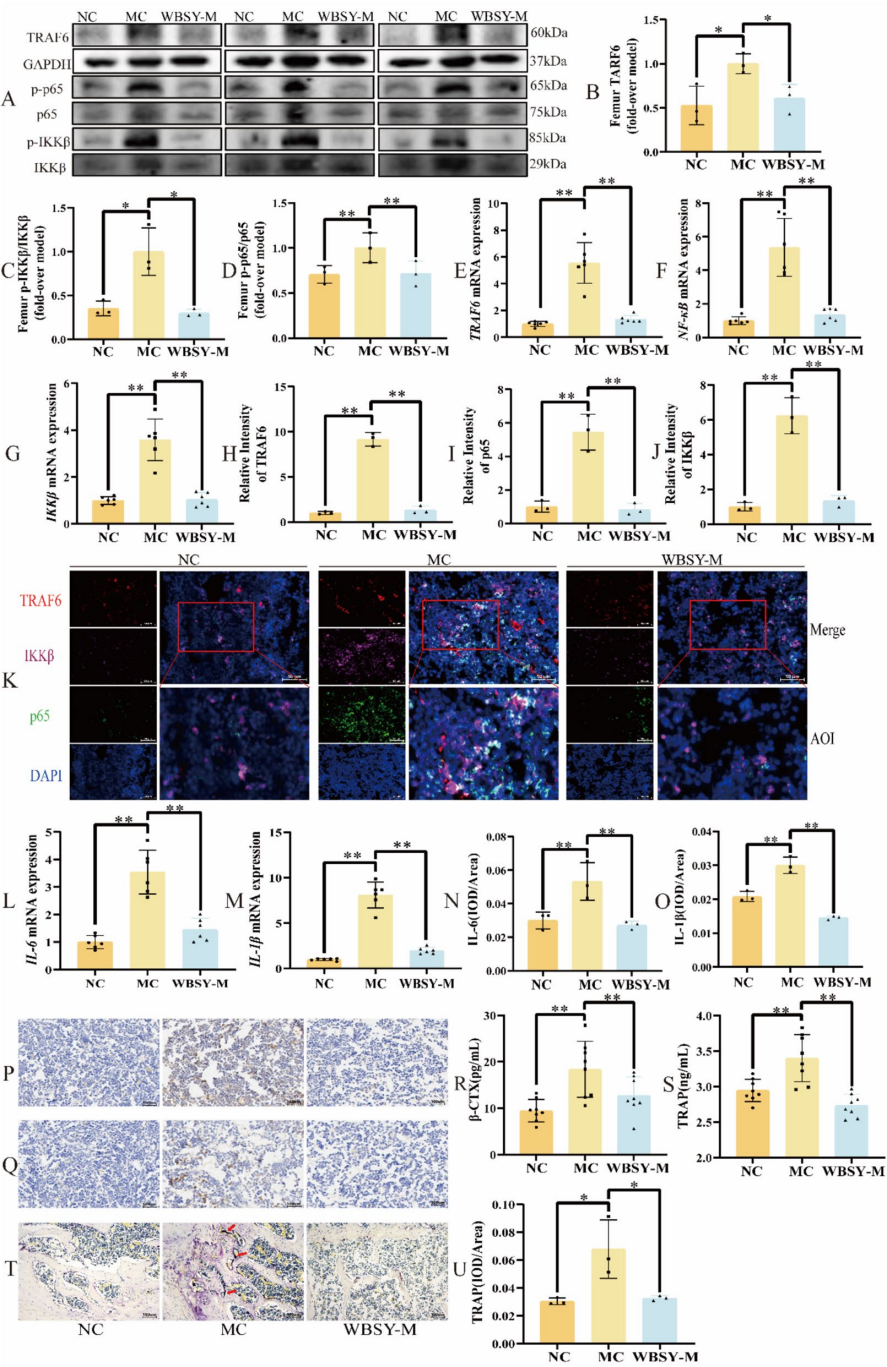
Effects of WBSY on bone resorption of model mice. The femur TRAF6-IKKβ-NF-κB Western blot representation (**A**) and protein expression statistical analysis (**B**–**D**), n = 3. The mRNA expression of *TRAF6*, *IKKβ* and *NF-κB* in the femur (**E**–**G**), n = 6. Immunofluorescence staining of TRAF6, IKKβ and NF-κB p65 in femur and femur representative microscopic images were counted by Image-J (400 ×) (**H**–**K**), n = 3. The mRNA expression of *IL-1β* and *IL-6* in the femur (**L**–**M**), n = 6. The expression of IL-1β and IL-6 in femur were observed by immunohistochemistry at 400 × magnification, and the IOD/Area was counted by Imagine Pro (**N**–**Q**), n = 3. Serum bone turnover markers β-CTX (**R**) and TRAP (**S**), n = 8. TRAP staining (SP × 200) of femur (**T**, **U**), n = 3. Data are expressed as the mean ± SD. ^*^p < 0.05 and ^**^p < 0.01 compare with the model control group

Building upon the previously described findings, we conducted a more in-depth investigation into the role and potential mechanisms of WBSY in the regulation of bone resorption. qRT-PCR and western blot analyses (Fig. 7A–G) revealed that, in comparison to the NC group, the mRNA and protein levels of TRAF6, NF-κB p65, and IKKβ were significantly elevated in the MC group (*p* < 0.05, *p* < 0.01). However, treatment with WBSY-M effectively inhibited these mRNAs and proteins (*p* < 0.05, *p* < 0.01). Immunofluorescence staining corroborated these findings, demonstrating a marked upregulation in the expression of TRAF6, IKKβ, and NF-κB p65 in the bone tissue of the MC group, an effect that was notably attenuated following WBSY-M intervention (*p* < 0.01), as shown in Fig. 7H–K.

In alignment with the activation results of these pathways, qRT-PCR analysis (Fig. 7L, M) revealed a significant upregulation in the mRNA expression levels of the pro-inflammatory cytokines *IL-1β* and *IL-6* in the MC group (*p* < 0.01). This finding was corroborated by immunohistochemical staining (Fig. 7N–Q), which demonstrated a marked increase in inflammatory cell infiltration within the bone tissue of the MC group (*p* < 0.01). Furthermore, enzyme-linked immunosorbent assay detection indicated a significant elevation in the serum levels of bone resorption markers β-CTX and TRAP in the MC group (*p* < 0.01). TRAP staining also revealed a substantial increase in osteoclast numbers (*p* < 0.05). Notably, these bone resorption abnormalities were significantly ameliorated following WBSY-M treatment (*p* < 0.05, *p* < 0.01) (Fig. 7R–U).

### Effects of WBSY on bone formation in model mice

**Fig. 8:**
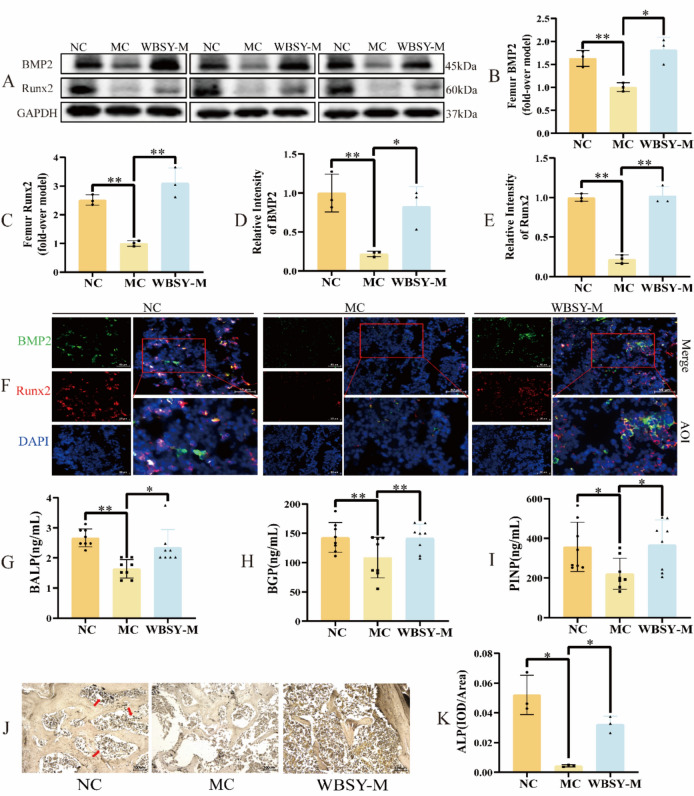
Effects of WBSY on bone formation of model mice. The femur BMP2-Runx2 Western blot representation (**A**) and protein expression statistical analysis (**B**, **C**), n = 3. Immunofluorescence staining of BMP2 and Runx2 in femur and femur representative microscopic images were counted by Image-J (400 ×) (**D**–**F**), n = 3. Serum bone turnover markers BALP (**G**), BGP (**H**) and PINP (**I**), n = 8. ALP staining (SP × 200) (**J**–**K**), n = 3. Data are expressed as the mean ± SD. ^*^p < 0.05 and ^**^p < 0.01 compare with the model control group

In addition to its role in inhibiting bone resorption, we further explored the regulatory function of WBSY in the bone formation process. Western blot analysis (Fig. 8A–C) demonstrated that the protein expression levels of BMP2 and Runx2, which are critical regulatory factors in bone formation, were significantly reduced in the femurs of mice in the MC group compared to those in the NC group (*p* < 0.01). However, treatment with WBSY-M significantly upregulated the expression of these proteins (*p* < 0.05, *p* < 0.01), restoring their levels to those comparable to the NC group. This alteration was further corroborated by immunofluorescence staining (Fig. 8D–F), which demonstrated a marked reduction in the fluorescence signals of BMP2 and Runx2 in the bone tissue of the MC group, while WBSY-M intervention significantly restored fluorescence intensity (*p* < 0.05, *p* < 0.01).

Additionally, the assessment of serum bone formation markers indicated that the levels of serum BALP, BGP, and PINP were significantly lower in the MC group compared to the NC group (*p *<0.05, *p* < 0.01), as described in Fig. 8G–I. Treatment with WBSY-M effectively reversed these declining trends (*p* < 0.05, *p* < 0.01). In addition, ALP staining (Fig. 8J) and quantitative analysis (Fig. 8K) of bone tissue showed a significant decrease in ALP activity in the MC group (*p* < 0.05), while the ALP activity significantly increased after WBSY-M treatment (*p* < 0.05), further confirming that WBSY can promote bone formation process.

## Discussion

WBSY is a Chinese patent medicine approved by the National Medical Products Administration. The *Pharmacopoeia of the People’s Republic of China (2025 Edition)* has clearly designated echinacoside, acteoside, and schisandrol as its key quality control components. Our study further verified that the contents and quality control requirements of these components in WBSY are in compliance with the provisions of the Pharmacopoeia. Previous studies have found that echinacoside, as one of the core components, can increase the total bone mineral density of the femur and the biomechanical strength of the tibia, and ameliorate osteoporosis induced by estrogen deficiency through the mechanism of promoting bone formation and inhibiting bone resorption [25, 26]. In addition, schisandrol modulates nuclear factor erythroid 2-related factor 2 to inhibit the production of reactive oxygen species induced by RANKL. Similarly, acteoside can inhibit osteoclast differentiation and maturation of osteoclast precursors by suppressing RANKL induced activation of mitogen-activated protein kinase (MAPK) and transcription factor NF–κB [23]. Concurrently, it suppresses the phosphorylation and nuclear translocation of p65 and prevents the degradation of IκBα, thereby mitigating bone loss in the ovariectomized osteoporosis model [34]. Collectively, modern pharmacological studies have confirmed that these components synergistically regulate bone metabolic balance through multiple pathways, laying a foundation for exploring WBSY’s therapeutic mechanisms in GIOP.

The dexamethasone-induced mouse GIOP model is an internationally recognized classic animal model of glucocorticoid-induced osteoporosis. The core rationale for selecting male C57BL/6 mice to establish the GIOP model in this study was to eliminate the confounding effects of cyclic fluctuations in estrogen levels during the estrous cycle of female mice on bone metabolism and immune function. As a critical regulator of bone homeostasis and T cell differentiation, the cyclic variations of estrogen in female mice would introduce significant experimental variability, which would interfere with the accurate assessment of the independent effects of glucocorticoids and WBSY [4, 51]. By using male mice, we were able to establish a more homogeneous and reproducible GIOP model, which allowed us to precisely dissect its immunomodulatory and bone metabolic regulatory mechanisms. Notably, our team has previously conducted a systematic study on the effects of WBSY in postmenopausal osteoporosis, confirming that it can promote osteoblast-mediated bone formation, inhibit osteoclast-mediated bone resorption, and significantly improve bone microstructure [16]. Therefore, this study specifically selected the GIOP model to simulate the clinically common drug-induced bone metabolism disorders, to further reveal the effects and mechanisms of WBSY against different types of osteoporosis, and promote its clinical translational research.

In this study, the clinically established anti-osteoporosis drug ALN was employed as the positive control. Its well-documented application and established efficacy in the clinical management of osteoporosis provide a reliable benchmark for assessing the validity of the research findings. Regarding key anti-osteoporosis indicators, such as bone formation and enhancement of bone microstructure, WBSY exhibited efficacy comparable to that of ALN, thereby preliminarily confirming its core therapeutic potential. Additionally, this study identified that in the dexamethasone-induced osteoporosis model, the model mice not only experienced the primary pathological change of bone loss but also exhibited weight loss and a significant decrease in anal temperature. Clinically, patients undergoing long-term glucocorticoid therapy may present with core skeletal issues, including bone mass loss and microstructural deterioration [44], alongside systemic functional impairments such as abnormal weight fluctuations, sensitivity to cold, and general fatigue [40]. The clinical manifestations observed are consistent with the phenotypic characteristics identified in the model mice utilized in this study. Our findings indicate that WBSY significantly ameliorates weight loss and the decline in anal temperature in these model mice. This research thus establishes a more clinically pertinent foundation for further investigation into the comprehensive intervention effects of WBSY on osteoporosis.

Bone microstructure serves as a fundamental indicator in the assessment of bone quality, playing a crucial and irreplaceable role in determining bone strength. In patients with osteoporosis, the degradation of trabecular morphology and connectivity constitutes the primary pathological feature associated with diminished bone strength, thereby markedly elevating fracture risk [46]. Dexamethasone, a widely utilized glucocorticoid in clinical settings, has emerged as a significant iatrogenic factor in bone metabolism disorders, particularly osteoporosis, resulting from prolonged use. The adverse impact of dexamethasone on bone microstructure represents a critical pathological mechanism contributing to the reduction in bone strength [55]. Previous research has established a strong correlation between the onset and progression of osteoporosis and aberrant alterations in critical parameters of bone trabeculae. These changes are primarily characterized by a reduction in trabecular number, a thinning of trabecular thickness, and diminished trabecular connectivity, alongside an increase in the structural model index and trabecular spacing. Notably, an elevated structural model index signifies a shift from normal plate-like trabecular structures to more fragile rod-like configurations, whereas an increased trabecular spacing results in larger interstitial gaps between trabeculae. These alterations collectively compromise the biomechanical integrity of bone tissue, ultimately leading to reduced bone strength [5]. In the present study, quantitative Micro Computed Tomography analysis revealed that the bone microarchitecture in a dexamethasone-induced osteoporosis mouse model exhibited substantial damage. This was evidenced by a marked reduction in bone volume/tissue volume ratio, with trabecular thickness and trabecular number displaying a dose-dependent decline, while structural model index and trabecular spacing significantly increased. Significantly, following the WBSY intervention, the previously mentioned pathological changes were effectively reversed. Subsequent biomechanical assessments corroborated this finding, demonstrating that the bone strength indicators in the WBSY intervention group mice were markedly superior to those in the model group. This suggests that the reparative effects of WBSY on bone microstructure ultimately result in enhanced bone strength.

To further elucidate the intrinsic molecular mechanisms of WBSY in counteracting GIOP, this study employed RNA sequencing analysis on mouse femoral tissue. The sequencing results indicated that, in comparison to the model group, the WBSY treatment group exhibited significant upregulation of 909 genes and significant downregulation of 1126 genes. This suggests that the dysregulation of these differentially expressed genes may be intricately linked to the pathogenesis of GIOP, and that their expression patterns can be effectively modulated through WBSY intervention. These findings provide critical insights for the subsequent identification of core target genes of WBSY. Furthermore, KEGG pathway enrichment analysis of the differentially expressed genes revealed significant enrichment in the IL-17 signaling pathway, osteoclast differentiation pathway, and chemokine signaling pathway. Notably, the IL-17 signaling pathway was the most significantly enriched immune-related pathway in GIOP mice, which formed the primary basis for our focus on the Th17/IL-17A axis as the core mechanism. Concurrently, we observed mild alterations in pathways associated with Th1 and Th2 cell function, which represent secondary accompanying changes in the GIOP pathological process. The aberrant IL-17 signaling pathway and chemokine signaling pathway are critical in modulating the equilibrium between bone immunity and bone metabolism. Their dysregulated activation can intensify bone resorption by promoting osteoclastogenesis and inhibiting osteoblast function. The enrichment of osteoclast differentiation pathways is directly linked to the fundamental pathological mechanism underlying bone metabolism imbalance. These findings suggest that WBSY is highly likely to exert its therapeutic effects against GIOP by targeting and modulating the activity of these pivotal signaling pathways.

In alignment with this, a growing body of evidence highlights the essential role of T cells in the development of GIOP. T cell-deficient severe combined immunodeficiency mice exhibit resistance to GIOP unless reconstituted with T cells, which subsequently increase serum RANKL levels and facilitate osteoclastogenesis [42]. Bone marrow-resident T cells express RANKL to potentiate osteoclast differentiation [24], a phenomenon replicated in RAW264.7 coculture systems [31]. Dexamethasone-induced immunosuppression paradoxically triggers CXCL10-CXCR3 pathway-mediated T cell homing to bone marrow [42]. CXCL10, via CXCR3 binding, recruits T cells to marrow niches, disrupting bone homeostasis. Enhanced CXCR3 expression exacerbates marrow T cell infiltration and bone remodeling dysfunction [21]. GIOP mice exhibit CXCL10/CXCR3 pathway activation with concomitant osteoclast activation and osteoblast suppression. CXCR3 knockout mice show attenuated inflammation and 50% reduced bone loss [42]. Our data demonstrate WBSY's capacity to normalize dexamethasone-suppressed splenic T cell/CD4^+^T cell counts, which may be related to the inhibition of CXCL10-CXCR3 activation and restoration of bone marrow T cell homeostasis.

In the bone marrow, overactivated T cells differentiate into Th17 cells under the regulation of RORγt, which are intricately linked to the pivotal regulatory pathways RANKL/RANK/OPG that govern osteoclast differentiation and immune regulation [11, 13]. Th17 cells have the capacity to directly produce RANKL or induce its expression in bone tissue by secreting IL-17. The stimulation of RANKL secretion by IL-17 further amplifies the effects of osteoclastogenesis [17, 58]. Additionally, IL-17A, derived from Th17 cells, can synergize with RANKL to enhance osteoclast activity and promote RANKL transcription by intensifying the pro-inflammatory cytokine cascade, thereby establishing a positive feedback loop that facilitates osteoclastogenesis [6]. Within this pathway, RANKL binds to RANK to promote osteoclastogenesis, whereas OPG serves as a competitive inhibitor, counteracting their interaction to maintain bone homeostasis [37, 60]. Numerous studies have demonstrated that GIOP and ovariectomy models corroborate a correlation between Th17 cell overactivation and elevated RANKL expression [2, 12, 61]. Both clinical and preclinical research consistently indicate an imbalance in the RANKL/OPG axis in osteoporosis, with OPG knockout mice exhibiting significant bone loss [6, 33]. The findings of this study further substantiate that dexamethasone can induce Th17 cell polarization in bone marrow, as evidenced by increased RORγt mRNA levels, while concurrently inhibiting OPG expression and upregulating RANKL/RANK expression, thereby disrupting the pathway balance. WBSY has been shown to mitigate Th17 cell overactivation by downregulating the mRNA levels of RORγt and IL-17A in bone tissue, reversing OPG inhibition, and reducing RANKL/RANK elevation, ultimately restoring the physiological balance of the RANKL/RANK/OPG signaling pathway.

In the downstream processes of the RANKL/RANK/OPG signaling pathway, the formation of the RANKL-RANK complex facilitates the recruitment of TRAF6, thereby activating the IKKβ-NF-κB signaling cascade, which is crucial for osteoclast maturation [8]. Osteoporotic models frequently demonstrate activation of the TRAF6-IKKβ-NF-κB pathway, leading to the nuclear translocation of NF-κB and the subsequent upregulation of proinflammatory cytokines such as IL-6 and IL-1β, which further exacerbate osteoclastogenesis [15, 36, 47]. In contrast, the BMP2-Runx2 signaling pathway plays a pivotal role in osteogenesis. Runx2, a master regulator of osteogenic differentiation, facilitates the commitment of mesenchymal stem cells to osteoblasts and promotes bone matrix mineralization. Additionally, BMP2 enhances Runx2-mediated bone regeneration, thereby counteracting the suppression of osteogenesis induced by RANKL [32]. Runx2^−/−^ mice are characterized by the presence of cartilaginous skeletons devoid of bone marrow [32], and osteoporotic models demonstrate a downregulation of BMP2 along with impaired osteogenesis [9]. Our findings are consistent with these observations: dexamethasone triggers the activation of the TRAF6-IKKβ-NF-κB pathway and suppresses BMP2-Runx2 expression in femoral tissues. Conversely, WBSY counteracts these effects by downregulating the TRAF6-IKKβ-NF-κB pathway and upregulating BMP2-Runx2 expression. Overall, these results substantiate the dual efficacy of WBSY in both reducing bone resorption and promoting osteogenesis through the coordinated regulation of critical signaling pathways.

To further validate WBSY’s effects on bone metabolism, we focused on classical biomarkers of bone resorption and formation. As a key enzyme secreted by osteoclasts, TRAP plays a central role in bone degradation and remodeling. Its activity directly correlates with osteoclast quantity and functional status, establishing TRAP as a classical biomarker for bone resorption assessment [14]. β-CTX, a specific marker derived from collagen breakdown, reflects bone resorption-formation imbalance when elevated [3, 7]. In vitro experiments demonstrated significantly enhanced TRAP activity in RAW 264.7 osteoclasts [22], while ovariectomy-induced bone loss mouse models exhibited proportional increases in TRAP and β-CTX levels with bone loss severity [50]. Our study revealed markedly elevated TRAP activity, β-CTX concentration, and TRAP protein expression in femoral tissues of model control mice, all of which were effectively reversed by WBSY intervention. Regarding bone formation, BALP facilitates osteogenesis by hydrolyzing inorganic phosphates to alleviate mineralization inhibition [49]. PINP, a specific product of bone matrix synthesis, dynamically reflects osteogenic activity through serum levels [28]. BGP regulates both bone matrix formation and resorption [35]. Chronic dexamethasone exposure significantly suppressed BALP, PINP, and BGP levels in model mice, impairing osteogenesis [39], consistent with our findings. Notably, WBSY treatment significantly modulated the levels of these biomarkers and enhanced the expression of ALP in femoral tissue. This finding corroborates its dual regulatory mechanism, which simultaneously inhibits bone resorption and promotes bone formation, thereby restoring homeostasis in bone metabolism.

From an indirect pharmacology perspective, this study delineates the indirect action pattern of WBSY against GIOP. Unlike the first-line anti-osteoporotic agent alendronate, which directly targets and inhibits osteoclasts, WBSY does not act directly on bone metabolic effector cells. Instead, it targets the bone immune system, with bone marrow T cells as the central intermediate unit. By modulating the CXCL10-CXCR3 axis to block aberrant T cell homing and subsequent IL-17A secretion, WBSY indirectly restores the RANKL/RANK/OPG pathway balance, achieving dual effects of inhibiting bone resorption and promoting bone formation. Nevertheless, we acknowledge that although our multi-omics and molecular experiments have confirmed WBSY's regulatory effects on these key nodes, the findings are largely based on correlative analyses, and direct causal evidence from functional validation experiments remains lacking. Furthermore, we have not performed in vitro cell experiments to verify the direct effects of WBSY and its main quality control components on CXCL10 secretion, CXCR3 expression, and T cell chemotactic migration, making it impossible to fully distinguish the direct immunomodulatory effects of the compound from the indirect effects secondary to improved bone metabolism. Future work will not only employ CXCR3 inhibitors, IL-17A neutralizing antibodies, and other targeted interventions to verify the causal role of bone marrow T cell homing in WBSY's anti-GIOP effects, but also systematically identify the cellular source of CXCL10 in the bone marrow and conduct in vitro functional validation of WBSY’s active components, providing more robust evidence for the clinical translation of traditional Chinese medicine in osteoporosis treatment.

## Conclusion

**Fig. 9:**
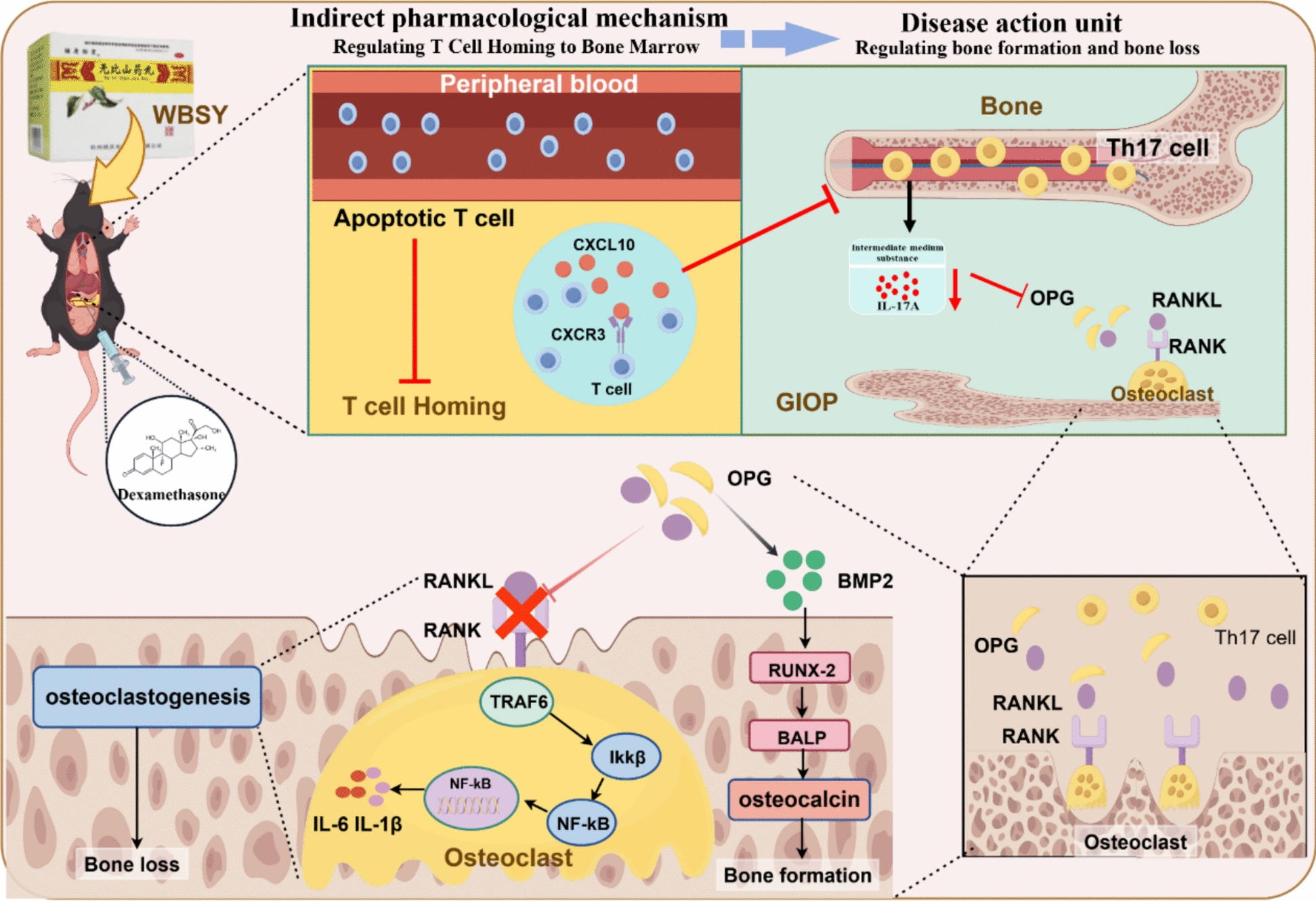
The mechanism of WBSY regulat bone marrow T Cell homing and mediate the RANK/RANKL/OPG Pathway to improve GIOP. This image was drawn on the figdraw website (https://www.figdraw.com) and has been certified with ID number SPTRP48285

In summary, this study suggests that WBSY exerts anti-GIOP effects via an indirect pharmacological mechanism, with bone marrow T cells serving as critical immune intermediates. WBSY may modulate the CXCL10-CXCR3 axis to indirectly inhibit aberrant T cell homing to the bone marrow, thereby reshaping the local immune microenvironment and preventing the excessive differentiation and activation of bone marrow-resident Th17 cells. This in turn reduces the secretion of the key pro-osteoclastogenic cytokine IL-17A, which acts as the critical molecular bridge linking immune dysregulation and bone metabolic disorder. This indirect immunomodulation subsequently restores the balance of the RANKL/RANK/OPG signaling pathway in bone tissue, leading to a synergistic promotion of bone formation and inhibition of bone resorption, as shown in Fig. 9.

## Data Availability

No datasets were generated or analysed during the current study.

## Abbreviations

ALN: Alendronate sodium
ALP: Alkaline phosphatase
BALP: Bone-specific alkaline phosphatase
BGP: Bone gla protein
BMP2: Bone morphogenetic protein 2
CXCL10: CXC chemokine ligand 10
CXCR3: Chemokine receptor 3
GIOP: Glucocorticoid-induced osteoporosis
IKKβ: IκB kinase β
IL-1β: Interleukin-1 Beta
IL-6: Interleukin-6
IL-17A: Interleukin-17A
NF-κB: Nuclear factor κB
OPG: Osteoprotegerin
PINP: Procollagen I N-terminal propeptide
p-IKKβ: Phosphorylated IκB kinase β
p-NF-κB: Phosphorylated nuclear factor κB
RANK: Receptor activator of nuclear factor-κB
RANKL: Receptor activator of nuclear factor-κB ligand
RORγt: Retinoic acid-related orphan receptor gamma t
Runx2: Runt-related transcription factor 2
TRAF6: TNF receptor-associated factor 6
TRAP: Tartrate-resistant acid phosphatase
WBSY: Wubi Shanyao Pill
β-CTX: ‌‌β-CrossLaps
